# Improving zinc accumulation in cereal endosperm using HvMTP1, a transition metal transporter

**DOI:** 10.1111/pbi.12749

**Published:** 2017-06-09

**Authors:** Paloma K. Menguer, Thomas Vincent, Anthony J. Miller, James K.M. Brown, Eva Vincze, Søren Borg, Preben Bach Holm, Dale Sanders, Dorina Podar

**Affiliations:** ^1^ The John Innes Centre Norwich Research Park Norwich UK; ^2^ Department of Molecular Biology and Genetics Faculty of Science and Technology Aarhus University Slagelse Denmark; ^3^ Department of Biology University of York York UK; ^4^ Faculty of Biology and Geology and Institute of Bionanotechnology Babeș‐Bolyai University Cluj‐Napoca Romania

**Keywords:** MTP, biofortification, mineral nutrition, membrane transporter, barley, SXRF

## Abstract

Zinc (Zn) is essential for all life forms, including humans. It is estimated that around two billion people are deficient in their Zn intake. Human dietary Zn intake relies heavily on plants, which in many developing countries consists mainly of cereals. The inner part of cereal grain, the endosperm, is the part that is eaten after milling but contains only a quarter of the total grain Zn. Here, we present results demonstrating that endosperm Zn content can be enhanced through expression of a transporter responsible for vacuolar Zn accumulation in cereals. The barley (*Hordeum vulgare*) vacuolar Zn transporter HvMTP1 was expressed under the control of the endosperm‐specific D‐hordein promoter. Transformed plants exhibited no significant change in growth but had higher total grain Zn concentration, as measured by ICP‐OES, compared to parental controls. Compared with Zn, transformants had smaller increases in concentrations of Cu and Mn but not Fe. Staining grain cross sections with the Zn‐specific stain DTZ revealed a significant enhancement of Zn accumulation in the endosperm of two of three transformed lines, a result confirmed by ICP‐OES in the endosperm of dissected grain. Synchrotron X‐ray fluorescence analysis of longitudinal grain sections demonstrated a redistribution of grain Zn from aleurone to endosperm. We argue that this proof‐of‐principle study provides the basis of a strategy for biofortification of cereal endosperm with Zn.

## Introduction

Zinc (Zn) is an essential micronutrient for all living organisms, performing both catalytic and structural roles in a wide variety of proteins. Almost 3000 human proteins, one tenth of the proteome, bind Zn (Andreini *et al*., 2006), but the number of protein–zinc interactions could be larger even than this (Maret, 2012). Thus, Zn is involved in regulating many important biological processes. While Zn toxicity in humans is very rare (Lu *et al*., 2009; Plum *et al*., 2010), Zn deficiency impairs development and growth, affects the nervous system, reduces immunity and can cause death. It is estimated that a third of the world population is suffering from moderate to severe Zn deficiency, while half of the population is at risk of being Zn deficient (Prasad, 2013; Wessells and Brown, 2012; WHO, 2002). Among micronutrient deficiencies, zinc deficiency ranks third, after iron and vitamin A deficiencies (WHO, 2013), that together account for almost half of the worldwide deaths of children under five (Black *et al*., 2013; WHO, 2013).

The primary cause of Zn deficiency in humans is poor nutrition because the main source of the metal ion is through dietary intake. Red meat comprises a principal dietary source of Zn, but in the most severely affected areas of dietary Zn deficiency, the population depends on staple grain crops such as wheat, maize, rice. These crops often contain low concentrations of zinc in the endosperm, the part of the kernel that is milled to make flour (Hansen *et al*., 2009; Lombi *et al*., 2011; Ozturk *et al*., 2006; Uddin *et al*., 2014). In most cases, low endospermal Zn is not due to growth in Zn‐poor soils (Fan *et al*., 2008), but rather to an uneven distribution of Zn within the tissues of the grain; although endosperm comprises up to two‐thirds of the grain biomass, it typically contains only 25% of grain Zn (Lombi *et al*., 2011). Bioavailability in the intestinal tract is also important for nutrition, and metal complexation influences absorption (Humer *et al*., 2015). Milling removes the embryo and the aleurone layers to leave flour containing less Zn than the whole grain (Cakmak, 2008; Cakmak *et al*., 2010; Ozturk *et al*., 2006). Cereal breeding generally has selected for traits such as high yield, disease resistance and starch quality rather than grain micronutrient content (Fan *et al*., 2008; Garvin *et al*., 2006; Zhao *et al*., 2009). Mineral analyses of currently cultivated cereal cultivars varies greatly, but generally the low concentrations within the grains is assumed to result from the dilution effect of increasing the yield (Garvin *et al*., 2006; White and Broadley, 2009, 2011; Zhao *et al*., 2009). Furthermore, wheat grain Zn concentration has been found to decrease with the date of release of the cultivar, suggesting that modern breeding is selecting against this trait (Fan *et al*., 2008; Garvin *et al*., 2006; Zhao *et al*., 2009).

Strategies to address Zn malnutrition have focused on soil fertilization, food fortification and dietary diversification, all of which – although efficient – are very difficult to achieve in developing countries where deficiency is most prevalent. A complementary intervention method is to breed crops with increased Zn content in the grain – so‐called biofortification (Borrill *et al*., 2014; Meenakshi *et al*., 2007; Palmgren *et al*., 2008; White and Broadley, 2005, 2009). Biofortification has been defined as the process of increasing the mineral status of staple crops within the edible parts through plant breeding. It ranks fifth among the intervention methods agreed by the Copenhagen Consensus (Center, 2008) to combat mineral malnutrition and it has proved to be highly cost‐effective (Bouis *et al*., 2011; Meenakshi *et al*., 2010). Biofortification requires no further investment once the crop lines with higher mineral content are identified and is thus more readily available to developing parts of the world.

To address Zn biofortification from a genetic perspective, the transporters involved in the grain deposition of Zn must first be identified and their metal specificities determined. In barley grains, Zn‐related transcriptome microarray analysis and expression studies in different grain tissues have identified several different metal ion transporter families: Heavy Metal ATPases (HMAs), Cation Diffusion Facilitators (CDFs, also known in plants as Metal Tolerance Proteins (MTPs)) and Natural resistance associated macrophage proteins (Nramps), that might be involved in the deposition of metals within the grain (Tauris *et al*., 2009). Plant MTPs are transition metal transporters that catalyse the efflux of Zn^2+^, Co^2+^, Fe^2+^, Cd^2+^, Ni^2+^ or Mn^2+^ from the cytoplasm to the outside of the cell or into subcellular compartments (Montanini *et al*., 2007). We have previously shown that the barley HvMTP1 transporter localizes to the vacuolar membrane, transports Zn to the vacuole when expressed in yeast and is specific for both Zn and Co, but not for other transition elements (Podar *et al*., 2012). The HvMTP1 transporter was also found to be expressed in phloem and aleurone cells and present but with low expression levels within transfer and endosperm cells of barley (Tauris *et al*., 2009). We hypothesized that over‐expression of *HvMTP1* on the strong endosperm‐specific D‐hordein promoter might provide a strategy to increase endosperm Zn content, thereby providing a proof of principle approach to cereal grain biofortification.

## Results

### Generating transformed plants expressing *HvMTP1* under D‐hordein promoter

**Figure 1:**
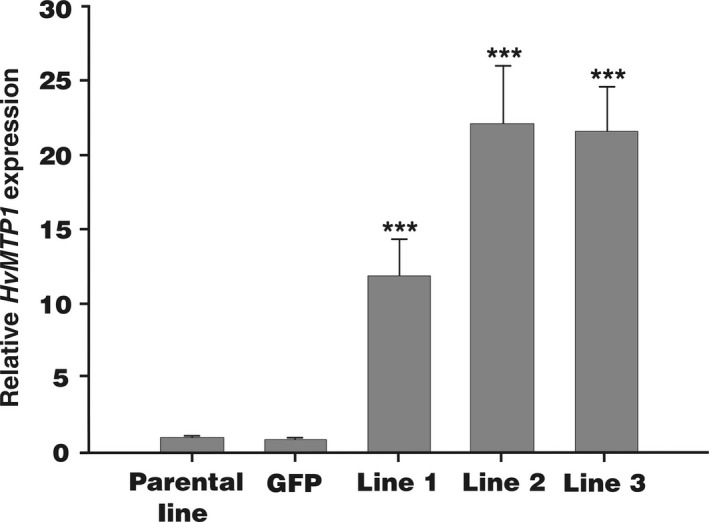
Quantitative real‐time RT‐PCR analysis of the relative expression levels of *HvMTP1* in grains of T6 barley plants, at 21 DAP. Expression of *HvMTP1* relative to *HvTUB2* is shown in parental lines, plants expressing *GFP* under the CaMV35S promoter (GFP) and transformed lines 1‐3 expressing *HvMTP1* under D‐hordein promoter. Plants were grown in the greenhouse in compost without addition of Zn. Bars represent means ± SE (*n* = 3). Values indicated with stars are statistically significant different from parental and GFP lines (analysis of variance; *P* < 0.001).

Transformed barley plants from a parental line (*cv*. Golden Promise) expressing *HvMTP1* under the endosperm‐specific D‐hordein promoter were generated to assess the role of HvMTP1 in grain metal accumulation. The barley D‐hordein promoter directs gene expression throughout the endosperm from 12 to 24 Days After Pollination: DAP (Furtado *et al*., 2009). Homozygous transformed plants with two (lines 2 and 3) and four copies (line 1) of *HvMTP1* were identified based on quantitative real‐time PCR (RT‐qPCR) of the hygromycin resistance gene. The Golden Promise parental line was used as a control, alongside a homozygous line constitutively expressing GFP under the CaMV35S promoter. RT‐qPCR analysis of the whole grain showed that the *HvMTP1* transcript was highly expressed at 21 DAP in all three independently transformed lines, while low levels of transcript were detected in both the parental and the *GFP*‐expressing lines. Data relating to *HvMTP1* endosperm‐specific promoter expression are shown for T6 grains (Figure 1). Transformed plants had similar germination rate, yield and morphology to the parental and GFP lines (Table S1 and Figure S1).

### HvMTP1 transformed plants retain whole grain Zn content

**Figure 2:**
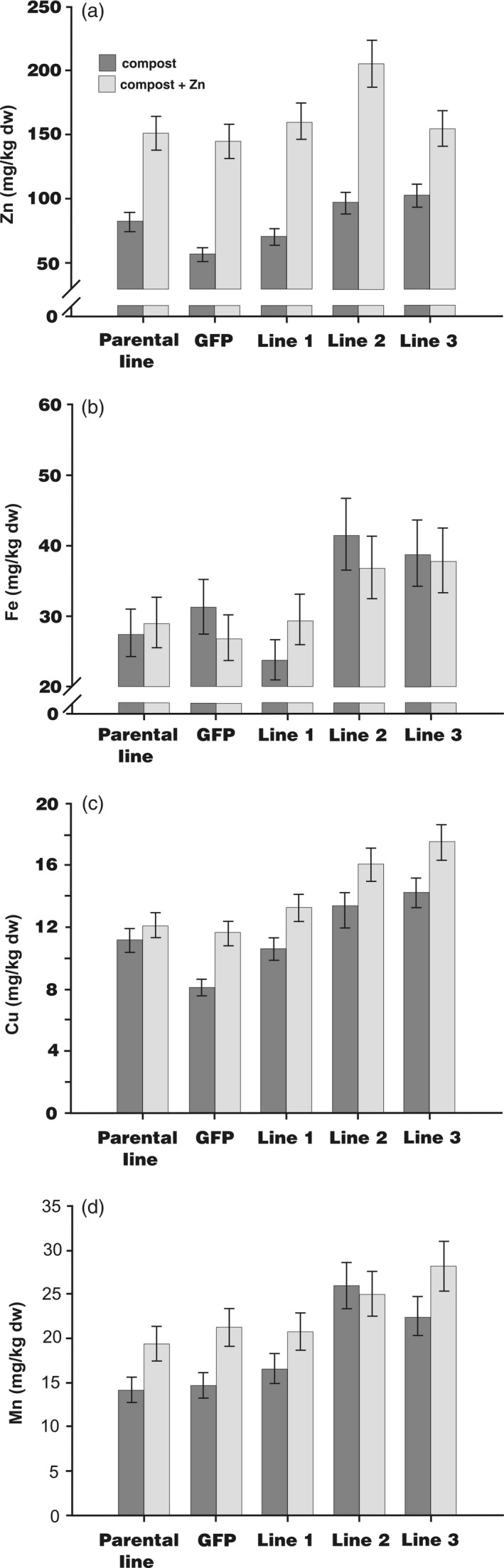
Concentrations of (a), zinc (Zn), (b), iron (Fe), (c), copper (Cu) and (d), manganese (Mn) in barley whole grains as measured by ICP‐OES in parental plants, plants expressing *GFP* under the CaMV35S promoter (GFP) and transformed lines 1‐3 expressing *HvMTP1* under the D‐hordein promoter. Plants were grown in the greenhouse in compost without (black) or with addition of 150 mg/kg ZnSO_4_ (grey). Bars represent means ± SE (*n* = 3) calculated by analysis of variance of log_10_ transformed data. Lines 2 and 3 had very significantly greater whole grain cation concentrations than the parental and *GFP*‐expressing plants (*P* < 0.001).

Barley plants were grown in pots with or without added ZnSO_4_ (150 mg/kg) until maturity to investigate whether enhanced *HvMTP1* expression in endosperm would increase grain Zn concentration. The three independent homozygous transformed lines and controls were examined for Zn accumulation in whole grain using ICP‐OES. The concentration of whole grain Zn of HvMTP1 transformed lines 2 and 3 was very significantly greater than in the parental or GFP lines (Figure 2a). Transformed lines 2 and 3 also showed significantly greater concentrations of whole grain Fe, Cu and Mn irrespective of the addition of Zn to the soil (Figure 2b, c, d). Supplementation of compost with Zn led to a mean 103% increase in the concentration of Zn in parental, GFP expressing and transformed HvMTP1 lines when compared to plants with no added zinc (*P* < 0.001; Figure 2a). There were smaller increases in the whole grain concentrations of Cu (*P* < 0.001; 24%; Figure 2c) and Mn (*P* < 0.001; 25%; Figure 2d) in Zn‐supplemented compost but no increase in Fe (*P* = 0.9; Figure 2b). There was no significant evidence that the parental, GFP or transformed lines differed from each other in the extent to which Zn supplementation affected concentrations of any of the four cations.

### Endosperm‐specific *HvMTP1* transformed plants exhibit higher accumulation of Zn in the endosperm

To determine the sites of Zn deposition within the grain, we deployed three independent approaches.

**Figure 3:**
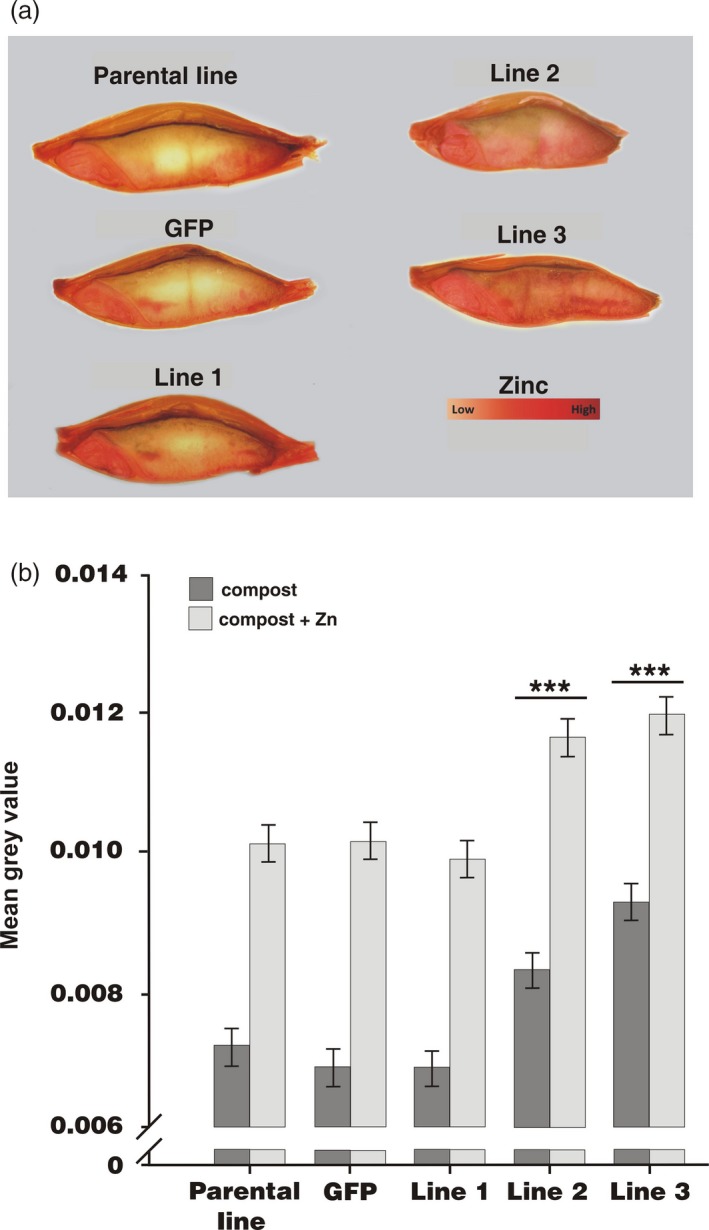
Distribution of Zn in barley grains shown through DTZ staining. (a) DTZ staining of grains grown in compost supplemented with 150 mg/kg ZnSO_4_. (b) Mean grey values of endosperm DTZ staining of parental line, plants expressing *GFP* under the CaMV35S promoter and transformed lines 1‐3 expressing *HvMTP1* under the D‐hordein promoter. Plants were grown in the greenhouse in compost without (black) or with addition of 150 mg/kg ZnSO_4_ (grey). Bars represent means ± SE (*n* = 15 with 2 technical replicates per grain). Lines indicated with three stars have mean grey values significantly different from those of the parental and GFP lines (analysis of variance; *P* < 0.001).

Longitudinal sections of grains were stained with diphenyl thiocarbazone (DTZ), which forms a red complex when bound to Zn and is highly selective for this metal, being interfered with only by Cd at high concentration (Ozturk *et al*., 2006). Figure 3 shows, as previously reported for wheat (Cakmak, 2008; Ozturk *et al*., 2006), that for the parental line, Zn is predominantly localized in the embryo and aleurone layer. A similar localization is apparent also for the *GFP*‐expressing control line, as well as for one of the *HvMTP1* expressing on the endosperm‐specific promoter lines (line 1). However, increased staining of Zn is visually apparent in the endosperm of the other two *HvMTP1* transformed constructs (lines 2 and 3; Figure 3a). Quantitative analysis of Zn staining within the endosperm alone indicated that lines 2 and 3 had very significantly higher levels of Zn compared to the parental and GFP lines with or without Zn supplementation, by a mean of 16% in line 2 and by 24% in line 3 (*P* < 0.001; Figure 3b).

**Figure 4:**
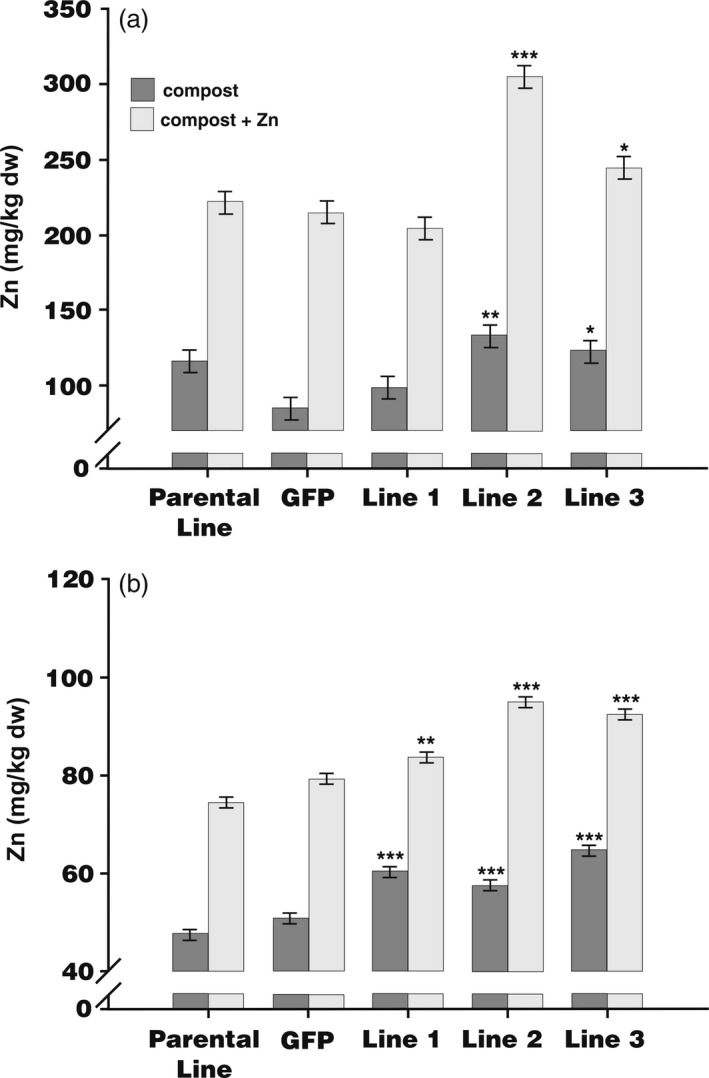
Concentration of zinc (Zn) in the (a), embryo plus bran layer and (b), endosperm (including the aleurone layers) as measured by ICP‐OES in the parental line, plants expressing *GFP* under the CaMV 35S promoter and transformed lines 1‐3 expressing *HvMTP1* under the D‐hordein promoter. Plants were grown in the greenhouse in compost without (black) or with addition of 150 mg/kg ZnSO_4_ (grey). Bars represent means ± SE (*n* = 3). Values indicated with stars are significantly different from those of parental and *GFP*‐expressing lines with or without added ZnSO_4_ as appropriate (analysis of variance; * 0.05 > *P* > 0.01, ** 0.01 > *P* > 0.001, *** *P* < 0.001).

To quantify Zn distribution, barley grains were dissected into endosperm (including the nutrient‐dense aleurone layers) and embryo plus bran layers and then analysed by ICP‐OES. Figure 4 shows that supplementation of compost with Zn led to an increase in the concentration of Zn in embryo and bran layer by at least twofold in all lines (Figure 4a). There was a significant increase in Zn accumulation in embryo plus bran layer comparing the endosperm‐expressing *HvMPT1* lines 2 and 3 with the parental and *GFP*‐expressing lines, regardless of whether or not Zn was supplemented. In general, there was also no consistent difference in Fe, Cu and Mn concentrations in both treatments for all plants (Figure S2).

Adding Zn to the growth medium similarly increased by at least 50% the concentration of Zn in the endosperm plus aleurone samples both in control and endosperm‐expressing *HvMTP1* lines (Figure 4b). Moreover, irrespective of Zn supplementation, the endosperm‐expressing *HvMTP1* lines showed increases of 9% to 31% of Zn concentration in the endosperm and aleurone compared with parental line, all three lines exhibiting statistically significant enhancement (Figure 4b). Cu and Mn accumulated in the endosperm, including the aleurone layer, of Lines 2 and 3 as did Fe in Line 1 (*P* < 0.001 in all cases; Figure S2a, c, e). There was also evidence for more variable accumulation of these cations in the embryo, specifically of Cu in Line 2 (*P* < 0.001; Figure S2d), Mn in Line 3 (*P* < 0.01; Figure S2f) and Fe in both these lines (*P* < 0.001; Figure S2b). These results strongly suggest that endosperm‐specific *HvMTP1* expression drives Zn redistribution within the grain increasing the concentration of Zn in the endosperm plus aleurone, but to a lesser extent in the whole grain. It may also enhance accumulation of other cations, especially in the endosperm.

To attain higher spatial resolution of distribution of Zn within the grain as a result of expression of *HvMTP1* on the endosperm‐specific promoter, Synchrotron X‐Ray Fluorescence (SXRF) mapping was performed on longitudinal grain sections of parental and transformed lines. All grains analysed were from plants grown without Zn supplementation.

**Figure 5:**
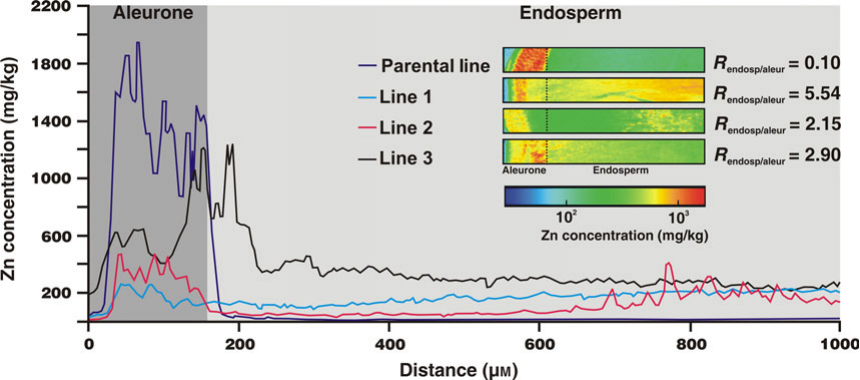
Grain line scans for Zn in parental line (purple) and transformed lines 1‐3 (blue, pink and black, respectively) expressing *HvMTP1* under D‐hordein promoter. Line scans begin on the outer margin of the grain (5 μm width) and continue 1000 μm towards the endosperm; data are displayed as Zn concentration in mg/kg. Distance 1–150 μm was assigned to the aleurone layers and 151–1000 μm to the endosperm. The insert shows μ‐XRF Zn maps of longitudinal sections of the grain (0.2 mm width and 1 mm length) in parental and transformed lines 1‐3. The colour scale represents different concentrations, with blue and red corresponding to the lowest and highest concentration, respectively. R, endosperm/aleurone ratio of areas under the line scan peaks calculated by Reimann sums.

Figure 5 demonstrates that in the parental line, Zn is principally distributed in the grain periphery, within a narrow band of approximately 150 μm which corresponds to the aleurone layer (Lombi *et al*., 2011). The Zn concentration decreases markedly beyond the point where the inner endosperm begins. By contrast, all three of the *HvMTP1* expressing lines have lower Zn in the aleurone layer, when compared to controls, in addition to an increased endosperm Zn signal (Figure 5). The endosperm/aleurone Zn concentration ratio increases from 0.1 in the parental line to 5.54, 2.15 and 2.90 in lines 1‐3. These results demonstrate that endosperm‐specific expression of *HvMTP1* leads to Zn grain redistribution from the aleurone layers into the endosperm. In contrast, the distribution of Fe, Ca and Cu is not affected by HvMTP1 overexpression (Figure S3). To facilitate sectioning of the barley grain a 12‐h presoak in water was used to soften the tissues. A parental line grain was also sectioned without soaking to analyse whether the water could be reallocating the minerals within the grain. The mineral distribution was similar for Zn, Fe, Ca and Cu, when comparing parental line either with or without water soaking (Figure S4). We therefore confirmed that presoaking the grain resulted in no significant metal redistribution within the grain.

## Discussion

Micronutrient acquisition through cereals is essential in many areas of the world, particularly in developing countries where meat consumption is not prevalent for reasons of sustainability or affordability (White and Broadley, 2011). Although whole grain itself might provide adequate dietary supply for humans, social and economic factors often determine – particularly in developing countries – that milled (endosperm‐rich) grain is eaten. Increased accumulation of Zn in cereal endosperm is therefore critical for human nutrition because this part of the grain is usually eaten: significant enhancement of Zn endosperm content could potentially contribute considerably to dietary Zn biofortification (Velu *et al*., 2014).

Within cereal grains Zn is mainly found in the pericarp, testa, embryo and aleurone, while the endosperm contains lower concentrations (Hansen *et al*., 2009; Lombi *et al*., 2011; Ozturk *et al*., 2006; Persson *et al*., 2009). Recent findings suggest that in barley (Uddin *et al*., 2014) and rice (Iwai *et al*., 2012), Zn can also be found in the outer endosperm in the subaleurone layers. Nonetheless, methods to develop high concentrations of Zn in the endosperm, which accounts for 60%–70% of the dry barley grain mass, are clearly desirable.

Strategies to increase the concentration of Zn in cereal grain at a most general level include agronomic practices such as the application of soil and foliar Zn fertilizers. The use of Zn fertilizers in deficient soils can increase grain yield and the accumulation of Zn by up to 300%, depending on the species and crop genotype (Graham and Rengel, 1993; Graham *et al*., 1992; Souza *et al*., 2014). In the current study, supplementation of compost with 150 mg/kg of ZnSO_4_ increased the concentration of Zn in grain by 103%, both in parental, GFP and endosperm overexpressing *HvMTP1* lines. There were much smaller increases in the accumulation of Cu and Mn while Fe was not affected. Nevertheless, Zn‐enriched soils or foliar‐applied sprays do not represent a sustainable agricultural solution in soils that are adequate for normal crop growth and development (Swamy *et al*., 2016; Wissuwa *et al*., 2008).

In addressing the issue of zinc biofortification, we have therefore taken a genetic rather than an agronomic approach. We reasoned that redistribution of Zn within cereal grains might provide a sustainable solution to endosperm enrichment of Zn. In this proof‐of‐principle study, we have been able to show through three separate approaches that increasing the expression of a vacuolar Zn transporter in cereal grain endosperm enhances accumulation of Zn in the endosperm. The HvMTP1 transporter chosen for these studies specifically transports Zn and cobalt, but not other metals (Podar *et al*., 2012).

All three of the techniques used here to assay the impact of HvMTP1‐specific expression in barley endosperm point to the promise of this approach. The qualitative overview provided by DTZ staining studies suggested enhancement of Zn uptake in two of three lines expressing HvMTP1 on an endosperm‐specific promoter. Using dissected grains, ICP‐OES provided quantitative data on enhanced endosperm Zn accumulation in HvMTP1 transformed lines. The quantitative SXRF analysis has provided spatially refined visualization and confirmation of our hypothesis that Zn can be redistributed from the aleurone to the endosperm with appropriate transporter arrangement. The correlation between increased *HvMTP1* transcript levels and Zn content in the endosperm strongly implies that HvMTP1 protein is up‐regulated in the endosperm and to a lesser extent in the embryo and bran.

Several transgenic approaches have been tested in recent years to increase cereal grain Zn accumulation. Overexpression in rice of the nicotianamine synthase genes *HvNAS1* under constitutive *Actin1* promoter (Masuda *et al*., 2009) or *OsNAS1* under the endosperm specific glutelin B1 promoter (Zheng *et al*., 2010) led to nearly 30% increase in grain Zn content, whereas *OsNAS2 and 3* almost doubled the amount of Zn accumulated in unpolished rice grains (Lee *et al*., 2009, 2011, 2012). Choice of promoters used to drive overexpression can impact the outcomes. Constitutive overexpression of the plasma membrane Zn transporters *AtZIP1* and *HvZIP7*, driven by the maize ubiquitin and double 35S promoters, increased the concentrations of Zn in barley grain by 60% and 35% (Ramesh *et al*., 2004; Tiong *et al*., 2014). However, in rice, constitutive overexpression of *OsZIP4* (35S promoter), *OsZIP5* (maize ubiquitin promoter) and *OsZIP8* (maize ubiquitin promoter) lowered Zn concentrations within the grains (Ishimaru *et al*., 2007; Lee *et al*., 2010a,b). What distinguishes the current study is the compartmental analysis of the Zn distribution within the grain. The use of the endosperm‐specific promoter has resulted in increases of up to 31% in Zn in the barley endosperm.

Major bottlenecks for Zn entering the endosperm are thought to be the translocation from the vascular tissue to the endosperm and the endosperm capacity to accumulate Zn (Ozturk *et al*., 2006; Stomph *et al*., 2011). Our results show that the limited capacity of the endosperm to store Zn can be controlled by sink demand.

Translation of these proof‐of‐principle findings to application will require bio‐availability studies of endosperm‐stored Zn. Phytate levels have conventionally been perceived to limit bioavailability of Zn through chelation (Palmgren *et al*., 2008). More recent studies, however, have suggested that sulphur (S) containing peptides rather than phytic acid bind to Zn in the barley embryo (Persson *et al*., 2009). The identity of the physiological ligands for Zn within the endosperm of cereals is still uncertain: in wheat endosperm, Zn was possibly associated with nicotianamine (Eagling *et al*., 2014; Xue *et al*., 2014) and did not correlate with S (Stomph *et al*., 2011); in rice Zn may also be associated with nicotianamine (Johnson *et al*., 2011); in barley endosperm, Zn was associated with S‐containing hordeins (Uddin *et al*., 2014).

It is therefore unclear from previous metabolite and co‐localization studies how Zn physiologically is bound in the grain, and what implications ligand binding might have for human nutritional bioavailability. We suggest here that generation of novel cereal germplasm with enhanced Zn endosperm accumulation offers new opportunities to explore the bottlenecks that limit grain Zn biofortification.

## Experimental procedures

### Vector construction

The zinc transporter HvMTP1 amplified with primers HvtMTP1_F and HvMTP1_R (Table S2) from barley (*Hordeum vulgare cv. Golden Promise*) seedlings (Podar *et al*., 2012) was cloned via *Xma*I in the pART7 vector (Gleave, 1992). The D‐hordein promoter (D‐HorP) was amplified with Phusion Hot Start High‐Fidelity DNA Polymerase (Finnzyme), using primers D‐HorP_F and D‐HorP_R (Table S2) from pHorGusNos vector and inserted via *Sac*I/*EcoR*I in pART7‐HvMTP1, thus constructing a *Not*I cassette that placed the HvMTP1 under the D‐hordein promoter and contained the ocs3′ terminator. The *Not*I cassette was further excised from pART7 and inserted in the pWBVec8 vector via *Not*I. Plasmids pWBVec8‐D‐HorP‐HvMTP1‐ocs3′T containing the construct in the inverted orientation with regard to the 35S promoter were selected and used for barley transformation. Plasmids were checked by sequencing after each cloning event.

### Plant transformation

Barley transformation was achieved using immature embryos infected with *Agrobacterium* strain AGL0 carrying the binary vector pWBVec8‐D‐HorP‐HvMTP1 (Matthews *et al*., 2001) and, as a control, pWBVec8‐35S‐GFP. After infection, embryos were transferred on *Agrobacterium* cocultivation, callus induction and subsequently, calli were placed on shoot induction and then root induction media. Primary putative transformants (T0) were rooted in the presence of hygromycin 50 mg/mL, in a growth room at 16‐h/8‐h light/dark cycle (light at 70 μmol/m^2^/s) and 24 °C/18 °C (day/night) temperature. Once rooted, plants from tissue culture were transferred to pots containing 3:1 Levingtons F2: Perlite and grown in a glasshouse with a minimum of 16‐h light (78 μmol/m^2^/s supplementary lighting for 12 h) and 20 °C/15 °C (day/night) temperature.

### Selection of transformants, segregation analysis and lines selection

Primary putative transformants (T1) were assessed for T‐DNA insertion both by PCR and leaf hygromycin resistance test (Wang and Waterhouse, 1997). PCR was performed using primers on D‐HorP_F and HvMTP1_137_R and GoTaq DNA Polymerase (Promega). Leaf hygromycin resistance test was performed on mature leaf segments placed on ½ MS media supplemented with hygromycin 200 mg/mL. Barley homozygous GFP expressing and parental lines were used as positive and negative controls. Only plants showing positive results for both the PCR and leaf resistance test were used for further generation analysis.

Grains of selected primary transformants were germinated (T2) and further assessed for segregation and single insertion by leaf resistance test. Plants with single insertion were expected to segregate according to Mendelian ratio of 3:1 hygromycin sensitive: resistant and therefore were selected for analysis. Grains of selected T2 plants constituted the T3 generation that was subjected to a second screening for zygosity by leaf resistance test. Only plants showing 100% resistance to hygromycin were retained and allowed to self‐pollinate. Grains were collected and germinated; the resulting plants represented the T4 generation. Genomic DNA of T4 plants was sent to iDNA Ltd Norwich for zygosity test and number of inserts based on quantitative PCR (RT‐qPCR) on hygromycin resistance gene.

Grains of T4 lines were also investigated for the level of expression of the zinc transporter by quantitative real‐time PCR (RT‐qPCR) using specific primers for *HvMTP1* and *HvTUB2* as a reference gene (Table S2). RNA was extracted (see RT‐qPCR section) from T4 grains at 21 days after pollination (21DAP), and 2 μg was used for cDNA preparation (SSIII reverse transcriptase Invitrogen). Lines with higher level of expression of HvMTP1 compared to control were selected for preliminary analysis of Zn levels within the endosperm by DTZ staining (Ozturk *et al*., 2006).

Three homozygous *D‐HorP‐HvMTP1* transformed lines showing 10‐ to 20‐fold higher level of expression of *HvMTP1* compared to parental background and potentially high levels of Zn within the endosperm was selected for further quantitative analysis: line 52c(4.1) with potentially two inserts and lines 30a(4.1) and 44(3.2) each with one copy of T‐DNA insertion, which were designated, for ease of reading, lines 1, 2 and 3, respectively.

### Analysis of selected transformed lines expressing *HvMTP1* under the D‐hordein promoter

#### Growth conditions

T5 generation of barley (*Hordeum vulgare* cv. Golden Promise) parental line and transformed lines (Ca35S_GFP and D‐hordein_HvMTP1 lines 1, 2 and 3) was grown in 2 L pots with or without Zn treatment. The compost mix consisted of Scotts Levington M2 compost, Perlite, Grit and Osmocote plant food (containing 0.02% Zn). Zinc treatment consisted of the addition of ZnSO_4_ (150 mg/kg) as a powder into the compost mix. Plants were grown in the greenhouse with no supplementary light regime, at temperatures of 15 °C day and 12 °C night. Individual spikes were tagged at flowering, and developing grains were harvested at 21 DAP when the barley D‐hordein promoter is highly active (Furtado *et al*., 2009). Samples were immediately frozen in liquid nitrogen and stored at −80 °C until RNA extraction for RT‐qPCR analysis. The remaining grains were harvested at maturity.

#### ICP‐OES analysis

Whole grain and dissected tissues sample were digested with a nitric acid (88%)/perchloric acid (12%) mix in a closed‐vessel microwave system (Milestone, Sorisole, Italy) then oven‐dried at 60 °C for 5 days. Metal concentrations (Cu, Fe, Mn Zn) were determined by inductively coupled plasma optical emission spectrometry (ICP‐OES; Vista‐Pro Axial; Varyan Pty Ltd, Mulgrave, Australia) at the School of Environmental Sciences, University of East Anglia. Reagent blanks, wheat flour certified reference material – NBS 1567a (from the US Department of Commerce National Bureau of Standards, Gaithersburg, MD 20899, USA) and standard metal solutions (Fluka) were used to assess the analytical quality control for sample preparation and the ICP‐OES. The whole grain analysis was performed in triplicate on 12 grains randomly collected from three different plants of each genotype.

Parental, GFP expressing and transformed lines (Ca35S_GFP and D‐hordein_HvMTP1 lines 1, 2 and 3) barley grains were carefully dissected by hand, after 24‐h soaking in water, using plastic tweezers and plastic razor blade under a dissecting microscope (Wild Heerbrugg, M3, Switzerland). To obtain a homogenous bulk grain sample, only grains weighing 34–45 mg were used for analysis. Parts of the grain were separated by dissection, the endosperm including the nutrient‐dense aleurone layers and embryo and bran layers, which were combined and analysed together. Dissected samples were oven‐dried at 60 °C for 5 days. Samples of tissues consisting of 12 grains collected from three different plants were analysed in triplicate.

#### Diphenylthiocarbazone staining of mature grains

Grains were placed in Milli‐Q water for two hours, before they were directly excised longitudinally along the crease with a scalpel. The sectioned grains were then added to 1.95 × 10^−3^ m DTZ dissolved in 100% methanol for 1 h, and the grains were then washed in water for 2 h (Ozturk *et al*., 2006). Next they were dried using medical wipes, before being placed on adhesive tape under a dissecting microscope. Grains were imaged using Qcapture software and analysed using ImageJ (Abramoff *et al*., 2004), in which they were converted to 8‐bit greyscale JPEGs and the mean grey value was calculated. The ‘polygon selections’ tool was used to select the endosperm for analysis. Five grains selected at random were analysed per genotype and this was repeated 3 times.

#### Quantitative RT‐qPCR analysis

Total RNA was isolated from tissues of developing grains at 21 DAP (containing high level of starch) as described previously (Li and Trick, 2005). The RNA pellets were washed carefully with 70% ethanol and resuspended in RNase free water followed by TURBO™ DNase (Applied Biosystems, Austin, TX) treatment. First‐strand cDNA synthesis was performed with oligo dT and reverse transcriptase (M‐MLV, Invitrogen, Carlsbad, CA) using 1 μg of RNA. Quantitative real‐time PCR (RT‐qPCR) reactions were performed in 96‐well plates in a CFX96 Touch™ Real‐Time PCR Detection System (Bio‐Rad, Hercules, CA) using 10 μL SYBR Green master mix (Life technologies, Carlsbad, CA), 5 μL of 1:20 diluted cDNA and 5 pmol of forward and reverse primers in a total volume of 20 μL. The *HvMTP1* was amplified from cDNA with HvMTP1_RT_F and HvMTP1_RT_R and as reference gene *HvTUB2* with HvTUB2_F and HvTUB2_R (Table S2). The standard thermal profile was 95 °C for 5 min, followed by 40 cycles of 95 °C for 30 s, 60 °C for 30 s and 72 °C for 30 s. Data were analysed using iQ5 Optical System software version 2.1 (Bio‐Rad, Hercules, CA). The comparative C_t_ (threshold cycle) method (Livak and Schmittgen, 2001) was applied to calculate the relative expression levels.

#### Synchrotron X‐ray fluorescence (SXRF)

Longitudinal sections (70 μm thick) of barley grains were obtained following the procedure described previously (Lombi *et al*., 2009), with a few modifications. Briefly, grains were soaked in water for 12 h to soften the samples for longitudinal sectioning using a razor blade. The flat longitudinal surface was glued to a metal support and sectioned using a vibrating blade microtome (VT1000S; Leica). A piece of Kapton polyimide film (DuPont, Eleutherian, Delaware, EUA) was pressed on the grain surface with the blade of the microtome cutting underneath to prevent the sliced section from crumbling apart. Two 70‐μm‐thick longitudinal sections from two independent grains (except for line 1 with only one section analysed) were analysed per sample using Synchrotron μ‐XRF at the Diamond Light Source, UK, on the I18 microfocus beamline. The incident X‐ray energy was set to 11 keV using a Si(111) monochromator. The X‐ray fluorescence spectra of Zn and other trace elements were collected using a Si drift detector. The beam size and step size were 5 μm with a dwell time of 0.2 s. Elemental quantification was carried out using an external calibration with XRF reference materials. Elemental distribution in longitudinal sections was very similar for Zn, and therefore, only one set of images for each grain type is presented in the paper (second set is presented in the Supplementary data). All grains analysed in this experiment are from plants grown in compost without Zn supplementation. Furthermore, a line scan across the grain, away from the embryo region, beginning on the dorsal outer margin (5 μm wide) and continuing 1000 μm towards the endosperm was recorded. The endosperm‐aleurone ratio integrates the areas under the peak (1–150 μm assigned to the aleurone layers and 151–1000 μm to the endosperm), calculated by Reimann sums, in parental and transformed lines.

### Statistical analysis

Data sets were analysed by general linear modelling, with standard errors calculated by analysis of variance and statistical significance tested by Fisher's protected least significant difference (GenStat 18th edition VSN International, Hemel Hempstead, UK).

## Authors' contribution

DP, DS and AJM conceived and designed the experiments. PKM, TV, AJM and DP performed the experiments. DP, EV, SB and PBH contributed to plant transformation. JKMB completed statistical analysis of the data. PKM, AJM, DS and DP wrote the manuscript.

## Supporting information


**Figure S1.** Representative plants.
**Figure S2.** Concentration of iron, copper and manganese in the endosperm and embryo.
**Figure S3.** μ‐XRF elemental maps of longitudinal sections of the grain of parental and HvMTP1 lines 1‐3.
**Figure S4.** μ‐XRF elemental maps of longitudinal sections of the grain of parental and HvMTP1 lines 2 and 3.
**Table S1.** Germination percentage of grains.
**Table S2.** List of primers.
