# Supplementary material for: Improving zinc accumulation in cereal endosperm using HvMTP1, a transition metal transporter
Source: Plant Biotechnol J. 2017 Jun 9;16(1):63–71. doi: 10.1111/pbi.12749 (PMC5785336; doi:10.1111/pbi.12749)
Supplement: Supplementary file 1 — Figure S1. Representative plants. Figure S2. Concentration of iron, copper and manganese in the endosperm and embryo. Figure S3. μ‐XRF elemental maps of longitudinal sections of the grain of parental and HvMTP1 lines 1‐3. Figure S4. μ‐XRF elemental maps of longitudinal sections of the grain of parental and HvMTP1 lines 2 and 3. Table S1. Germination percentage of grains. Table S2. List of primers. [file PBI-16-63-s001.doc]

**Supplementary information**

**Figure S1.** Representative plants grown in the greenhouse in soil without addition of Zn. From left to right: parental line plant, plant expressing *GFP* under the CaMV35S promoter and plants of transformed lines 1-3 expressing *HvMTP1* under the D-hordein promoter (a). Yield of plants grown in the greenhouse in soil without addition of Zn (b). Bars represent means ± SE (n=3). There were no significant differences between HvMTP1 transformed, parental and GFP lines.

**Figure S2.** Concentration of iron (Fe) (a, b), copper (Cu) (c, d) and manganese (Mn) (e, f) in endosperm including the aleurone layers (a, c, e) and embryo plus bran layer (b, d, f) measured by ICP-OES in the parental line, plants expressing *GFP* under the 35S promoter and transformed lines 1-3 expressing *HvMTP1* under the D-hordein promoter. Plants were grown in the greenhouse in compost without (black) or with addition of 150 mg kg^-1^ ZnSO_4_ (grey). Bars represent means ± SE (n=3). Values indicated with stars are significantly different from those of parental and *GFP*-expressing lines with or without added ZnSO_4_ as appropriate (analysis of variance; ** 0.01>P>0.001, *** P<0.001).

**Figure S3.** μ-XRF elemental maps of longitudinal sections of the grain (0.2 mm width and 1 mm length; grain dorsal side below) in parental line and transformed lines 1-3 expressing *HvMTP1* under D-hordein promoter. The colour scale represents different concentrations, with blue and red corresponding to the lowest and highest concentration, respectively. All grains were soaked in water for 12 h before sample sectioning and imaging.

**Figure S4.** μ-XRF elemental maps of longitudinal sections of the grain (0.2 mm width and 1 mm length; grain dorsal side below) in parental line and transformed lines 2-3 expressing *HvMTP1* under D-hordein promoter. The colour scale represents different concentrations, with blue and red corresponding to the lowest and highest concentration, respectively. All grains were soaked in water for 12 h before sample sectioning and imaging. The parental line was also sectioned without previous soaking for elemental maps comparison.

**Supplementary figures**

**Figure S1**

**
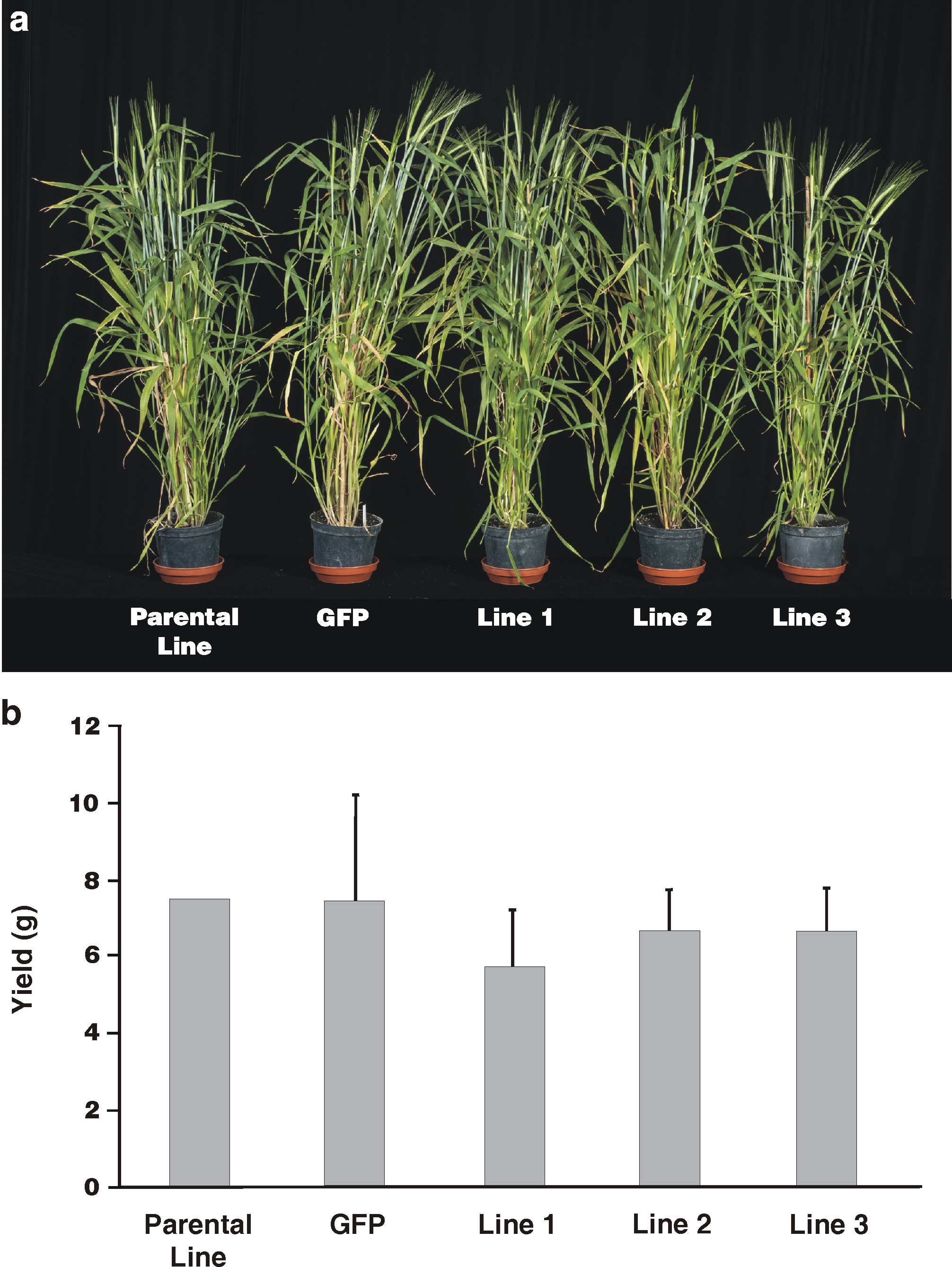
**

**Figure S1.** Representative plants grown in the greenhouse in soil without addition of Zn. From left to right: parental line plant, plant expressing *GFP* under the CaMV35S promoter and plants of transformed lines 1-3 expressing *HvMTP1* under the D-hordein promoter (a). Yield of plants grown in the greenhouse in soil without addition of Zn (b). Bars represent means ± SE (n=3). There were no significant differences between HvMTP1 transformed, parental and GFP lines.

**Figure S2**

**
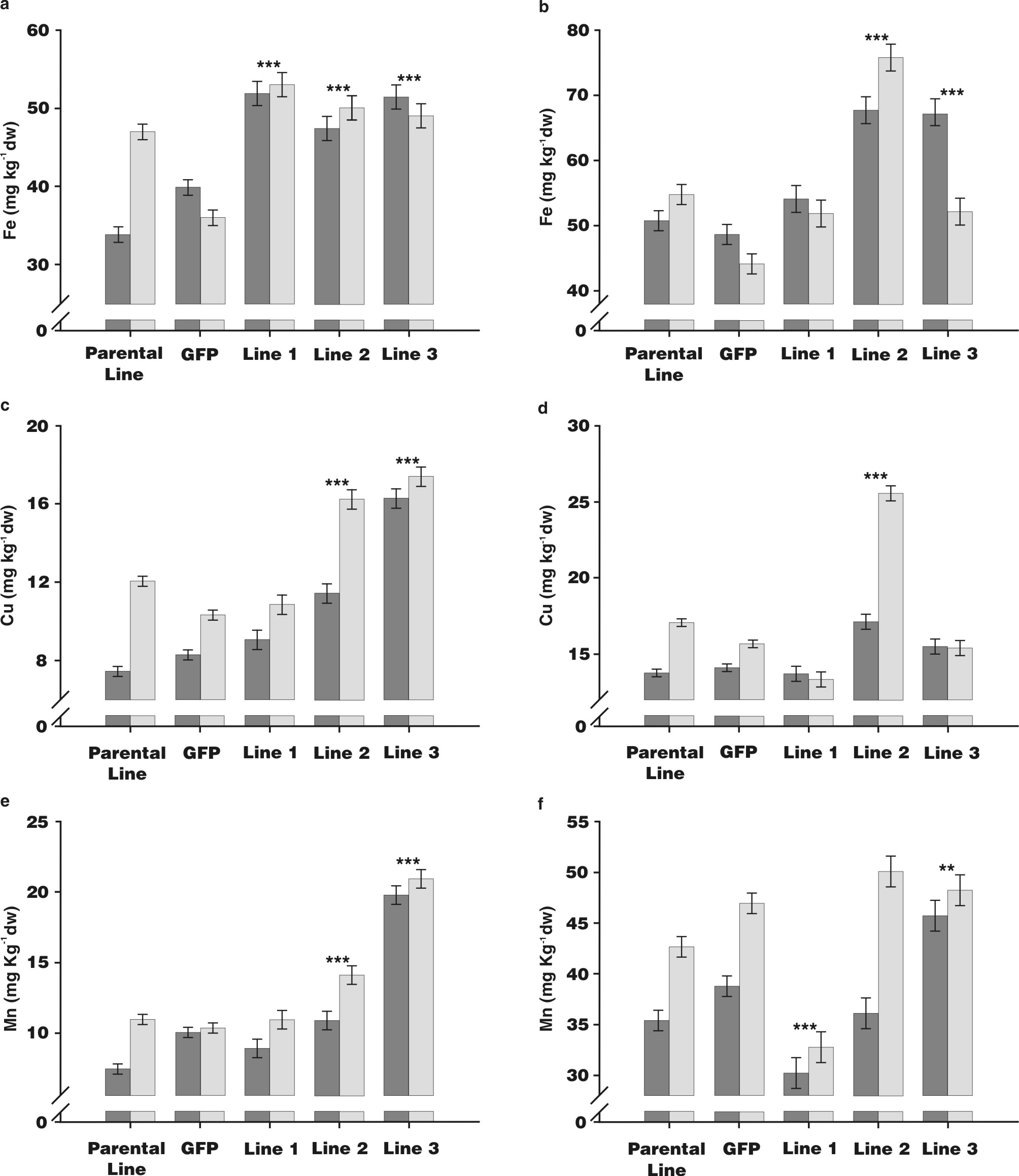
**

**Figure S2.** Concentration of iron (Fe) (a, b), copper (Cu) (c, d) and manganese (Mn) (e, f) in endosperm including the aleurone layers (a, c, e) and embryo plus bran layer (b, d, f) measured by ICP-OES in the parental line, plants expressing *GFP* under the 35S promoter and transformed lines 1-3 expressing *HvMTP1* under the D-hordein promoter. Plants were grown in the greenhouse in compost without (black) or with addition of 150 mg kg^-1^ ZnSO_4_ (grey). Bars represent means ± SE (n=3). Values indicated with stars are significantly different from those of parental and *GFP*-expressing lines with or without added ZnSO_4_ as appropriate (analysis of variance; ** 0.01>P>0.001, *** P<0.001).

**Figure S3**

**
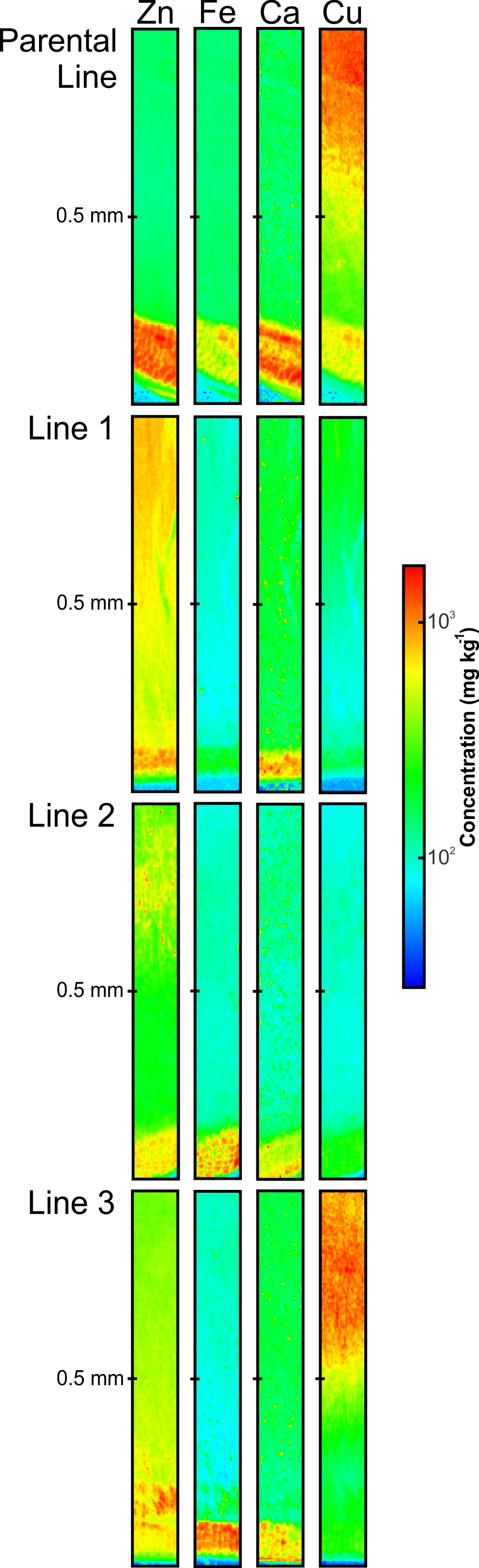
**

**Figure S3.** μ-XRF elemental maps of longitudinal sections of the grain (0.2 mm width and 1 mm length; grain dorsal side below) in parental line and transformed lines 1-3 expressing *HvMTP1* under D-hordein promoter. The colour scale represents different concentrations, with blue and red corresponding to the lowest and highest concentration, respectively. All grains were soaked in water for 12 h before sample sectioning and imaging.

**Figure S4**

**
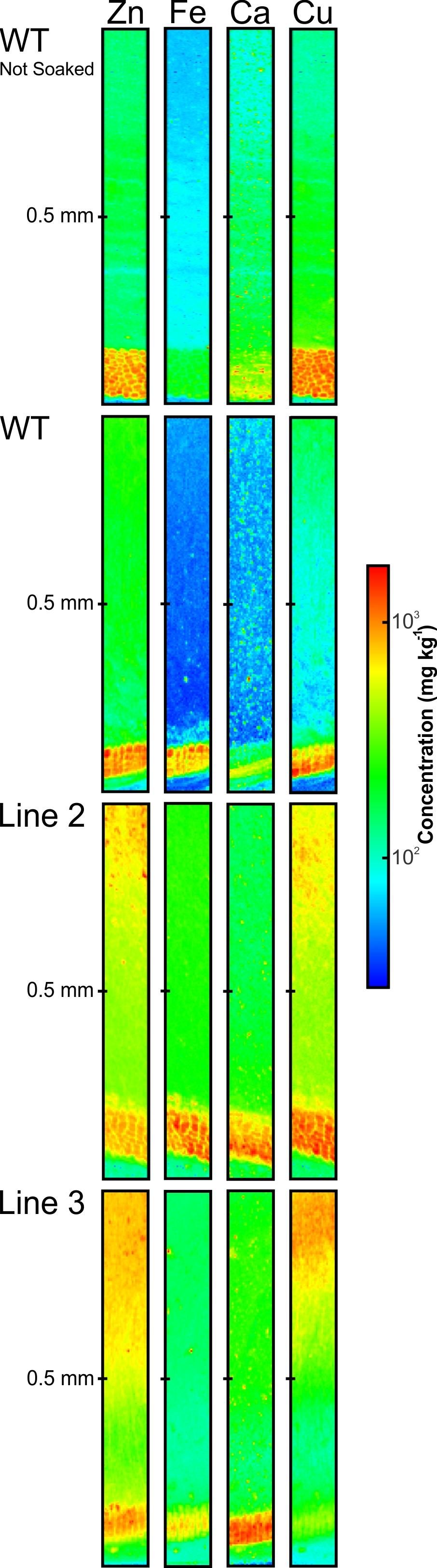
**

**Figure S4.** μ-XRF elemental maps of longitudinal sections of the grain (0.2 mm width and 1 mm length; grain dorsal side below) in parental line and transformed lines 2-3 expressing *HvMTP1* under D-hordein promoter. The colour scale represents different concentrations, with blue and red corresponding to the lowest and highest concentration, respectively. All grains were soaked in water for 12 h before sample sectioning and imaging. The parental line was also sectioned without previous soaking for elemental maps comparison.

**Table S1. Germination percentage of grains from parental, GFP expressing *GFP* under CaMV35S promoter and transformed lines 1-3 expressing *HvMTP1* under D-hordein promoter. Grains were collected from plants grown in soil without or with addition of Zn.**

|  | **Day 3 (germination %)** | **Day 6 (germination %)** |
| --- | --- | --- |
| **Parental** | **86** | **86** |
| **GFP** | **80** | **90** |
| **Line 1** | **100** | **100** |
| **Line 2** | **90** | **93** |
| **Line 3** | **90** | **100** |
| **Parental + Zn** | **90** | **90** |
| **GFP + Zn** | **83** | **86** |
| **Line 1 + Zn** | **93** | **93** |
| **Line 2 + Zn** | **90** | **96** |
| **Line 3 + Zn** | **83** | **86** |

**Table S2: List of primers used for cloning *HvMTP1* and D-Hordein promoter, and for quantitative real-time PCR.**

| **Gene** | **Direction** | **Sequence (5’-3’)** |
| --- | --- | --- |
| HvMTP1 | sense | taaacccgggatggacagccataattcatcaccg |
| HvMTP1 | antisense | taaacccgggttactctcgctcaatctgaatggtcac |
| HvMTP1_137 | antisense | tctttcgaggtgttaccggcatcag |
| D-HorP | sense | tcccgagctcgcggccgcgcttcgagtgcccgccgatttg |
| D-HorP | antisense | taaagaattcctcggtggactgtcaatgaattg |
| HvMTP1_RT | sense | ggagccaagcctgctaagaa |
| HvMTP1_RT | antisense | ggagccaagcctgctaagaa |
| HvTUB2 | sense | gcttgacaatgaggccatctatg |
| HvTUB2 | antisense | tgtatgttgggcgctcaatg |
| HvMTP1 | sense | taaacccgggatggacagccataattcatcaccg |
| HvMTP1 | antisense | taaacccgggttactctcgctcaatctgaatggtcac |
